# Jasmonic acid deficiency leads to scattered floret opening time in cytoplasmic male sterile rice Zhenshan 97A

**DOI:** 10.1093/jxb/erx251

**Published:** 2017-07-26

**Authors:** Li Liu, Zhengshan Zou, Ke Qian, Chan Xia, Ying He, Hanlai Zeng, Xie Zhou, Michael Riemann, Changxi Yin

**Affiliations:** 1 College of Plant Science and Technology, Huazhong Agricultural University, Wuhan, China; 2 College of Life Sciences, Nanjing Agricultural University, Nanjing, China; 3 Botanical Institute, Molecular Cell Biology, Karlsruhe Institute of Technology (CS), Kaiserstr, Karlsruhe, Germany

**Keywords:** cytoplasmic male sterile rice, filament, floret opening time, jasmonic acid, lodicule, osmotic regulation substances, potassium, soluble sugar, water accumulation

## Abstract

Cytoplasmic male sterile (CMS) rice has been widely used for hybrid rice seed production in China. However, CMS rice suffers from undesirable flowering habits including scattered floret opening time (FOT), which causes different FOTs among parental rice plants and greatly reduces hybrid rice seed production. Little is known about the mechanism of scattered FOT in CMS rice. Our results demonstrate that scattered FOT in CMS rice Zhenshan 97A (ZS97A) resulted from the lack of a driving force to open florets, which was directly caused by retarded lodicule expansion. Our results indicate that retarded lodicule expansion in ZS97A was caused by reduced water accumulation due to retarded accumulation of osmotic regulation substances (ORSs). Further, the retardation in accumulation of ORSs and water were caused by jasmonic acid (JA) deficiency, resulting from down-regulation of *OsAOC* expression. Applying JA restored scattered FOT in ZS97A by promoting ORS and water accumulation, and inducing the expansion of the lodicules. Taken together, JA deficiency inhibited lodicule expansion by retarding the accumulation of ORSs and water, leading to scattered FOT in CMS rice ZS97A.

## Teaser

Jasmonic acid deficiency in the lodicule leads to scattered floret opening time in cytoplasmic male sterile rice by retarding lodicule expansion through reduced accumulation of osmotic regulation substances and water.

## Introduction

Rice (*Oryza sativa* L.) is a major staple food that feeds more than half of the human population (Delseny *et al.*, 2001). Hybrid rice takes advantage of heterosis (hybrid vigor), which has been successfully utilized in many countries, leading to 10–20% yield increase over inbred varieties (Cheng *et al.*, 2007). Thus, cultivating hybrid rice is an effective approach to significantly increase grain yield (Zhang *et al.*, 2013). However, during hybrid rice seed production, floret opening time (FOT) of almost all cytoplasmic male sterile (CMS) rice is scattered, whereas FOT of male fertile rice (maintainer and restorer lines) is centralized, with all florets opening during a particular time of day. The different FOTs among parental rice plants generally lead to a low rate of cross-pollination and low yield of hybrid rice seed production.

In gramineous plants, simultaneously the filaments extend and the lodicules swell rapidly immediately before flowering (Heslop-Harrison and Heslop-Harrison, 1996). Rice is a typical gramineous plant, with six filaments and a pair of lodicules inside each floret. Just before the rice floret opens, the filaments elongate rapidly and the lodicules at the base inside the floret swell rapidly (Hoshikawa, 1989). As a result, the glume (lemma and palea) is levered away, and the floret opens. Thus, the extending filaments and/or swelling lodicules may provide the driving force to open the floret by levering away the glume, and CMS rice plants with a scattered FOT may lack the driving force to open florets.

Jasmonic acid (JA) and its derivative jasmonates are important plant hormones involved in plant responses to biotic and abiotic stresses, root growth, sex determination, leaf senescence, and fruit ripening (He *et al.*, 2002; Wang *et al.*, 2002; Song *et al.*, 2013, 2014; Ye *et al.*, 2013; Guo *et al.*, 2014; Riemann *et al.*, 2015; Yuan and Zhang, 2015; Jia *et al.*, 2016; Liu *et al.*, 2016). In addition, jasmonates play important roles in regulating floret development, filament elongation, and lodicule swelling and withering (Cheng *et al.*, 2009; Cai *et al.*, 2014; Xiao *et al.*, 2014).

The action of JA requires its biosynthesis and signaling. Many developmental processes and stress responses are impaired in JA-deficient and signaling mutants (Sanders *et al.*, 2000; Stintzi and Browse, 2000; von Malek *et al.*, 2002; Riemann *et al.*, 2013; Ye *et al.*, 2013; Guo *et al.*, 2014; Hazman *et al.*, 2015; Lee *et al.*, 2015; Dhakarey *et al.*, 2016). In the dicotyledonous model plant Arabidopsis, the filament elongation is inhibited both in JA-deficient mutants, including *aos*, *opr3*, *dde2-2* and *lox3lox4*, and in the JA signaling mutant *coi1* (Feys *et al.*, 1994; Stintzi and Browse, 2000; Park *et al.*, 2002; von Malek *et al.*, 2002; Caldelari *et al.*, 2011). In the monocotyledonous model plant rice, the FOT of JA-resistant mutant *osjar1* is scattered and the *osjar1* florets open at random hours and also during the night instead of in a certain period of the daytime as with wild-type florets (Xiao *et al.*, 2014). Moreover, our previous reports revealed that exogenous jasmonates strongly promote floret opening in CMS rice, and that the floret opening response to jasmonates in CMS rice was more sensitive than that of male fertile rice (Zeng *et al.*, 1999; Song *et al.*, 2001). These results suggested that JA plays a critical role in regulating FOT in rice, and that CMS rice florets may be JA deficient. In addition, it has been reported that CO_2_ induces rice floret opening by promoting lodicule expansion via the ‘acid growth effect’ (Wang *et al.*, 1989); however, the promoting effect of jasmonates on opening of rice florets does not depend on the ‘acid growth effect’ (Zeng *et al.*, 1999).

These results suggest that scattered FOT in CMS rice may result from the lack of a driving force to open the florets, which may be due to JA deficiency in CMS rice florets. However, this suggestion lacks strong evidence, and the mechanism of scattered FOT of CMS rice remains elusive. In order to explore this mechanism, we investigate the lack of a driving force to open the florets, the cause of JA deficiency, and the underlying mechanism of JA deficiency on FOT using an elite CMS rice, Zhenshan 97A (ZS97A) (*Oryza sativa* L. *indica*), and its isonuclear maintainer line, Zhenshan 97B (ZS97B) (*Oryza sativa* L. *indica*).

## Materials and methods

### Plant materials and growth conditions

ZS97A and its isonuclear maintainer line ZS97B were used in this study. ZS97A is the female parent of a number of widely used hybrids in China. ZS97A and ZS97B have an identical nuclear background but different cytoplasms. They normally have no significant phenotypic differences at the vegetative stage, but FOT in ZS97A is scattered at the flowering stage, whereas that of ZS97B is centralized.

All rice plants were grown according to Yin *et al.* (2007). ZS97A and ZS97B seeds were immersed in distilled water for 2 d, grown for 1 month in a greenhouse and transplanted to an experimental paddy field.

### Chemicals and treatments

JA and ibuprofen (IBU) were purchased from Sigma-Aldrich (Shanghai) Trading Co., Ltd. They were dissolved in a small amount of ethanol and diluted to the desired concentration with deionized water. Control water received the same amount of ethanol alone, but without JA or IBU. The pH values of all treatment solutions were adjusted to 6.5 with 0.1 M HCl or NaOH.

Panicles having florets nearing anthesis, in which several florets had flowered before the experimental date, were selected for tests. The selected panicles were carefully immersed in 100-ml flasks containing different treatment solutions for 30 s. The effects of the treatments on lodicule volume, lodicule fresh and dry weights, lodicule water content, filament length and potassium and total soluble sugar (TSS) contents were monitored.

### Measurements of filament length and lodicule volume

The lemma and palea were peeled away with forceps before photographing the filament and lodicule using a stereoscopic microscope (Leica S8 APO). The length of the filament, and the length, width and height of the lodicule were measured with ImageJ. Lodicule volume was calculated using the ellipsoid volume formula: *V*=4/3π*abc*; where *V* is volume, π is 3.14, *a* is half-length, *b* is half-width, and *c* is half-height.

### Measurement of lodicule fresh and dry weights

Samples with >50 pairs of lodicules were removed and collected from the florets, and the fresh weight of each sample was measured immediately using an analytical balance with readability of 0.1 mg. The samples were oven dried at 105 °C for 30 min and then dried at 80 °C to constant weight. The dry weight of each sample was measured, and the fresh and dry weights of each pair of lodicules were calculated.

### Determination of potassium and TSS contents

Each sample with 100 pairs of lodicules was removed and collected from the florets. The potassium content of each sample was determined by atomic absorption spectrometry following the method of Ieggli *et al.* (2010). The TSS content of each sample was determined using the anthrone colorimetric method reported by Zhang *et al.* (2011).

### JA content analysis

Lodicules were collected and stored at −80 °C. The samples were prepared using a modified crude extraction procedure originally reported by Pan *et al.* (2008). The JA contents of the lodicule samples were analysed using ultra-fast liquid chromatography–electrospray ionization tandem mass spectrometry according to the method of Liu *et al.* (2012).

### RNA isolation, reverse transcription–polymerase chain reaction (RT-PCR) and quantitative RT-PCR analyses

Total RNA was extracted from lodicules using an RNAprep Pure Plant Kit (Tiangen Biotech, China) following the instructions in the user manual. First-strand cDNA was synthesized from 1 μg of total RNA using a FastQuant RT Kit (Tiangen Biotech, China).

As shown in Table 1, 13*OsLOX* genes, four *OsAOS* genes, one *OsAOC* gene, ten *OsOPR* genes, and three *OsACX* genes in rice have been reported previously (Ohta *et al.*, 1992; Haga and Iino, 2004; Kim *et al.*, 2007; Tani *et al.*, 2008; Marla and Singh, 2012; Riemann *et al.*, 2013; Huang *et al.*, 2014). However, *OsLOX3* (LOC_Os03g49260), *OsAOS3* (LOC_Os02g12680), and *OsAOS4* (LOC_Os02g12690) are not involved in JA biosynthesis in rice (Kuroda *et al.*, 2005; Mizuno *et al.*, 2003). Thus, expression levels of the JA biosynthetic genes, including 12 *OsLOX*, two *OsAOS*, one *OsAOC*, ten *OsOPR* and three *OsACX* genes between the ZS97A and ZS97B lodicules were compared using reverse transcription–polymerase chain reaction (RT-PCR) and quantitative RT-PCR (qRT-PCR).

**Table 1.** Information on *LOX, AOS, AOC, OPR*, and *ACX* family genes in rice

Gene name in this study	MSU_Locus	Alternative name	Reference
*OsLOX1*	LOC_Os02g10120		Marla and Singh (2012)
*OsLOX2*	LOC_Os03g08220		Marla and Singh (2012)
*OsLOX3*	LOC_Os03g49260	*r9-LOX1*, *Osr9-LOX1*	Mizuno *et al.* (2003); Marla and Singh (2012); Zhou *et al.* (2014); Xu *et al.* (2015)
*OsLOX4*	LOC_Os03g49350		Marla and Singh (2012)
*OsLOX5*	LOC_Os03g49380	*OsLOX1*	Wang *et al.* (2008); Marla and Singh (2012);
*OsLOX6*	LOC_Os04g37430		Marla and Singh (2012)
*OsLOX7*	LOC_Os05g23880		Marla and Singh (2012)
*OsLOX8*	LOC_Os08g39850		Marla and Singh (2012)
*OsLOX9*	LOC_Os08g39840	*RLL*, *OsHI-LOX*	Peng *et al.* (1994); Zhou *et al.* (2009); Marla and Singh (2012)
*OsLOX10*	LOC_Os11g36719		Marla and Singh (2012)
*OsLOX11*	LOC_Os12g37260		Marla and Singh (2012)
*OsLOX12*	LOC_Os12g37350		Marla and Singh (2012)
*OsLOXL-2*	LOC_Os03g52860	*L-2*, *OsLOX2*	Ohta *et al.* (1992); Huang *et al.* (2014)
*OsAOS1*	LOC_Os03g55800		Haga and Iino (2004)
*OsAOS2*	LOC_Os03g12500		Haga and Iino (2004)
*OsAOS3*	LOC_Os02g12680	*OsHPL2*	Haga and Iino (2004); Kuroda *et al.* (2005)
*OsAOS4*	LOC_Os02g12690	*OsHPL1*	Haga and Iino (2004); Kuroda *et al.* (2005)
*OsOPR1*	LOC_Os06g11290	*OsOPR2*, *OsOPR11*	Sobajima *et al.* (2003); Tani *et al.* (2008)
*OsOPR2*	LOC_Os06g11280		Tani *et al.* (2008)
*OsOPR3*	LOC_Os06g11260		Tani *et al.* (2008)
*OsOPR4*	LOC_Os06g11240	*OsOPR10*	Tani *et al.* (2008)
*OsOPR5*	LOC_Os06g11210	*OsOPR6*	Tani *et al.* (2008)
*OsOPR6*	LOC_Os06g11200	*OsOPR4*	Tani *et al.* (2008)
*OsOPR7*	LOC_Os08g35740	*OsOPR3*, *OsOPR5*, *OsOPR9*, *OsOPR13*	Tani *et al.* (2008); Guo *et al.* (2014)
*OsOPR8*	LOC_Os02g35310	*OsOPR7*	Tani *et al.* (2008)
*OsOPR9*	LOC_Os01g27240		Tani *et al.* (2008)
*OsOPR10*	LOC_Os01g27230	*OsOPR12*	Tani *et al.* (2008)
*OsAOC*	LOC_Os03g32314	*cpm2*, *hebiba*	Agrawal *et al.* (2003); Riemann *et al.* (2003); Riemann *et al.* (2013)
*OsACX1*	LOC_Os06g01390		Kim *et al.* (2007)
*OsACX2*	LOC_Os11g39220		Kim *et al.* (2007)
*OsACX3*	LOC_Os06g24704		Kim *et al.* (2007)

The expression levels of JA biosynthetic genes were analysed by RT-PCR using the primers listed in Supplementary Table S1 at *JXB* online. The rice *OsACTIN* (NM_001057621) gene fragment was used as an internal control in the RT-PCR analysis. The *OsACTIN* fragment was amplified at 94 °C for 3 min, followed by 24 cycles at 94 °C for 45 s, 56 °C for 45 s, 72 °C for 45 s, and an extension at 72 °C for 10 min. The *OsLOX1*, *OsLOX2*, *OsLOX4*, *OsLOX5*, *OsLOX6*, *OsLOX7*, *OsLOX8*, *OsLOX9*, *OsLOX10*, *OsLOX11*, *OsLOX12*, *OsLOXL-2*, *OsOPR2*, *OsOPR3*, *OsOPR4*, *OsOPR5*, *OsOPR6*, *OsOPR8*, *OsOPR9*, and *OsOPR10* fragments were amplified at 94 °C for 3 min, followed by 38 cycles at 94 °C for 45 s, 56 °C for 45 s, 72 °C for 45 s, and an extension at 72 °C for 10 min. The *OsAOS2*, *OsOPR1*, *OsOPR7*, *OsACX1*, *OsACX2*, and *OsACX3* fragments were amplified at 94 °C for 3 min, followed by 36 cycles at 94 °C for 45 s, 56 °C for 45 s, 72 °C for 45 s, and an extension at 72 °C for 10 min. The *OsAOS1* fragment was amplified at 94 °C for 3 min, followed by 32 cycles at 94 °C for 45 s, 56 °C for 45 s, 72 °C for 45 s, and an extension at 72 °C for 10 min. The *OsAOC* fragment was amplified at 94 °C for 3 min, followed by 27 cycles at 94 °C for 45 s, 56 °C for 45 s, 72 °C for 45 s, and an extension at 72 °C for 10 min.

The JA biosynthetic gene expression levels were also determined by qRT-PCR analysis on an iQ5 Real-Time PCR Detection System (Bio-Rad, USA). The primers used for qRT-PCR are listed in Supplementary Table S2. The relative expression level of target genes was calculated using the comparative threshold (*C*_T_) method, with *OsACTIN* as the internal control.

### Statistical analysis

Statistical analysis was performed using an independent samples *t*-test, or one-way ANOVA followed by Duncan’s multiple range test with at least three replicates. Values of *P*<0.05 were considered statistically significant. All data were expressed as means±SE.

## Results

### FOT is scattered in CMS rice ZS97A

**Fig. 1.**
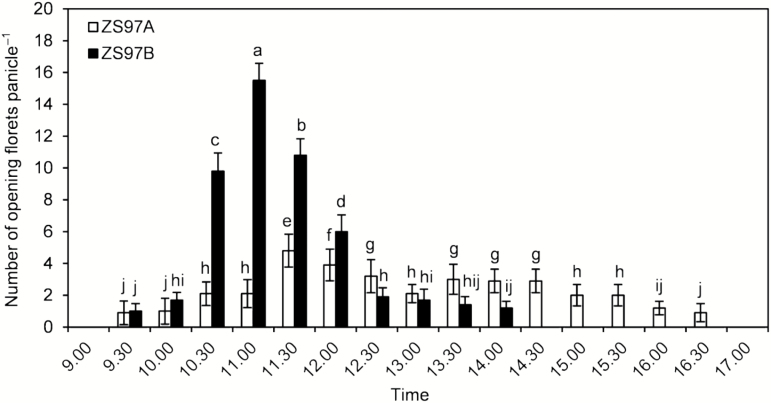
Comparison of floret opening time (FOT) between ZS97A and ZS97B panicles. Panicles having florets nearing anthesis, in which several florets had flowered before the experimental date, were selected for tests. The number of florets opening on selected panicles was recorded at 09.00, 09.30, 10.00, 10.30, 11.00, 11.30, 12.00, 12.30, 13.00, 13.30, 14.00, 14.30, 15.00, 15.30, 16.00, 16.30, and 17.00 h. Data are presented as means±SE. Standard errors of the number of opening florets per panicle were calculated from ten biological replicates. Significant differences (*P*<0.05) are indicated by different letters.

ZS97B florets opened from 09.30 to 14.00 h (Fig. 1). Flowering clearly peaked in ZS97B; 70.78% of the ZS97B florets opened from 10.30 to 11.30 h, and 87.84% before 12.00 h (Fig. 1). By contrast, the ZS97A florets opened from 09.30 to 16.30 h (Fig. 1). Only 25.71% of the ZS97A florets opened from 10.30 to 11.30 h, and 42.29% opened before 12.00 h (Fig. 1). These results demonstrate a scattered FOT in CMS rice ZS97A.

### Scattered FOT is associated with retarded lodicule expansion in ZS97A

**Fig. 2.**
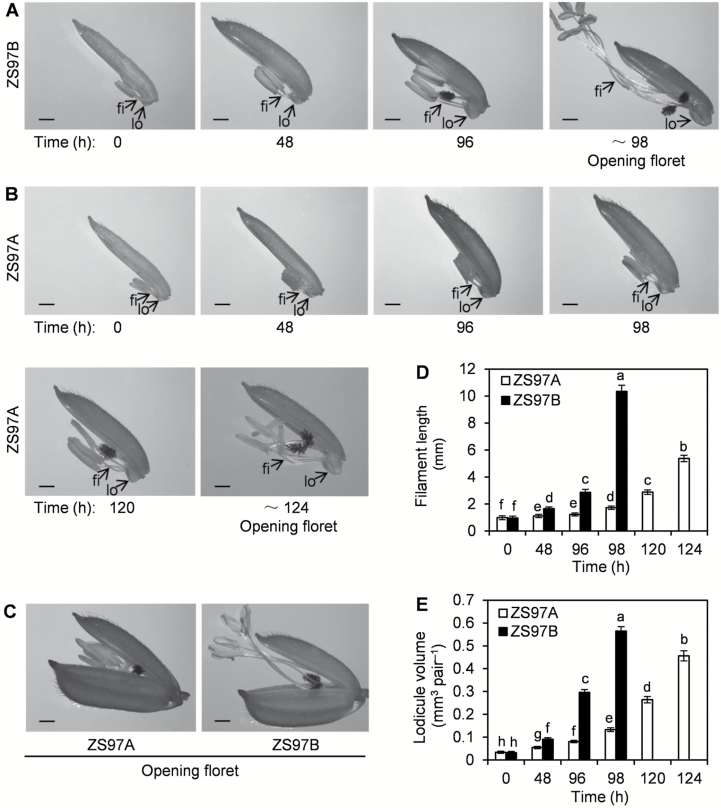
Comparison of filament extension and lodicule expansion between ZS97A and ZS97B florets. The investigation began at 10.00 h (0 h), 4 d before heading, and ended at floret opening time (FOT). The upper florets on ZS97A and ZS97B panicles were collected after 0 h (the beginning of investigation), 48, 96, and 98 h from the beginning of the investigation, and the upper florets on ZS97A panicles were collected after 120 and 124 h from the beginning of the investigation. Filament length and lodicule volume of the collected florets were measured immediately after sampling, and photographs were taken at the same time. (A) Dynamic changes in filament length and lodicule size of ZS97B panicles. (B) Dynamic changes in filament length and lodicule size of ZS97A panicles. (C) Opening florets of ZS97A and ZS97B. (D) Length of filaments of ZS97B florets. (E) Lodicule volume of ZS97B florets. Data are presented as means±SE (D, E). Standard errors of filament length and lodicule volume were calculated from 20 and 10 biological replicates, respectively (D, E). fi, filament; lo, lodicule. Scale bars (A–C): 1 mm. Significant differences (*P*<0.05) are indicated by different letters (D, E).

Immediately before the floret opening, the filaments of ZS97B florets extended rapidly, and the fully elongated 10.34 mm-long filament pushed the anther out of the glume (Fig. 2C, D). However, extension of the filaments in ZS97A florets was delayed and inhibited, and their final length was only 5.38 mm, and thus the shorter filaments failed to push the anther out of the glume (Fig. 2C, D). Moreover, lodicule expansion was delayed and inhibited in ZS97A florets. The expansion period of the upper florets on ZS97A and ZS97B panicles lasted for ~124 h and ~98 h, respectively, from 10.00 h 4 d before heading to FOT (Fig. 2A, B, E). The volume of fully expanded lodicules of ZS97A florets was smaller than that of ZS97B florets (Fig. 2A, B, E). This result demonstrates that filament extension and lodicule expansion were delayed and inhibited in ZS97A florets.

Filaments extended and the lodicules swelled rapidly and simultaneously immediately before floret opening in ZS97B (Fig. 2A, D, E), suggesting that the extending filaments and/or swelling lodicules might provide the driving force to open the floret by levering away the glume. Thus, retarded floret opening may have been caused by the retardation of filament extension and/or lodicule expansion in ZS97A.

**Fig. 3.**
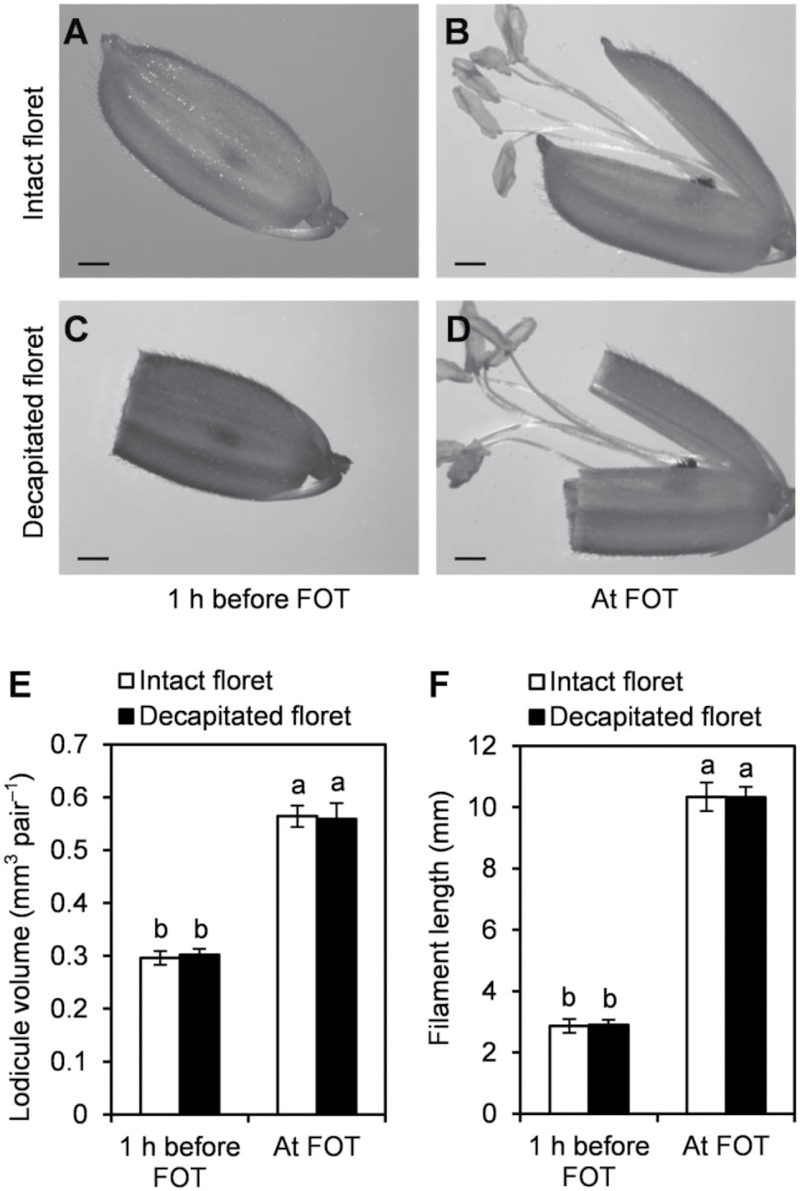
Effect of decapitation (upper part of glumes were decapitated) on filament extension, lodicule expansion, and floret opening. At 10.00 h, ZS97B florets that will open in 1 h were selected for the investigation, and half of the selected florets were decapitated. (A) Intact floret at 10.00 h (1 h before floret opening time (FOT)). (B) Intact floret at 11.00 h (FOT). (C) Decapitated floret 1 h before FOT. (D) Decapitated floret at FOT. (E) Lodicule volume of ZS97B floret 1 h before FOT and at FOT. (F) Filament length of ZS97B floret 1 h before FOT and at FOT. Scale bars (A–D): 1 mm. fi, filament; lo, lodicule. Data are presented as means±SE (E, F). Standard errors of filament length and lodicule volume were calculated from 20 and 10 biological replicates, respectively (E, F). Significant differences (*P*<0.05) are indicated by different letters (E, F).

The driving force for floret opening was investigated in ZS97B florets to test whether scattered FOT was caused by retarded filament extension and/or lodicule expansion in ZS97A. As shown in Fig. 3C, D, the upper parts of ZS97B glumes were decapitated 1 h before FOT, and the extending filament did not promote floret opening by levering away the glume. However, the filament extended and the lodicule swelled normally even though the upper parts of the glumes were decapitated (Fig. 3A–D). The length of the filaments and volume of the lodicules increased 3.57- and 1.85-fold, respectively, beginning 1 h before FOT until FOT (Fig. 3E, F). Because the upper parts of the glumes were decapitated and the extending filament did not promote floret opening by levering away the glume, floret opening may be driven by swelling lodicules. This result suggests that swelling lodicules rather than extending filaments were the main driving force to open the florets by levering away the glumes. Therefore, although filament extension as well as lodicule expansion were retarded in ZS97A florets, retarded lodicule expansion, rather than retarded filament extension, led to a lack of driving force, which caused scattered FOT in ZS97A.

### Retarded lodicule expansion is correlated with the retardation of water accumulation in ZS97A lodicules

**Fig. 4.**
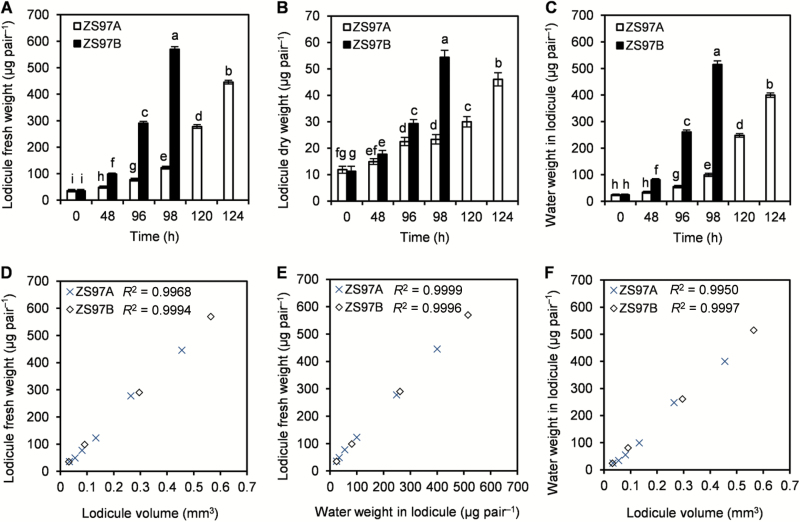
Relationships between lodicule fresh weight, lodicule dry weight, water weight in lodicule, and lodicule volume. The investigation began at 10.00 h (0 h), 4 d before heading, and ended at floret opening time (FOT). The lodicules from the upper florets on ZS97A and ZS97B panicles were collected after 0 h (the beginning of the investigation), 48, 96, and 98 h from the beginning of the investigation, and the lodicules from the upper florets on ZS97A panicles were collected after 120 and 124 h from the beginning of the investigation. Lodicule fresh weight, lodicule dry weight, water weight in lodicule, and lodicule volume of the samples were measured from 0 h to FOT. (A–C) Dynamic changes in lodicule fresh weight, lodicule dry weight and water weight in lodicule. (D) Relationship between lodicule fresh weight and lodicule volume. (E) Relationship between lodicule fresh weight and water weight in lodicule. (F) Relationship between water weight in lodicule and lodicule volume. Data are presented as means±SE (A–C). Standard errors of lodicule fresh and dry weights, and water weight in lodicule were calculated from three biological replicates. Significant differences (*P*<0.05) are indicated by different letters (A–C).

Fresh weights of the ZS97A and ZS97B lodicules were 36.03 and 35.31 μg pair^−1^, respectively, at the beginning of the investigation (10.00 h, 4 d before heading day), whereas their fresh weights increased to 445.70 and 569.50 μg pair^−1^, respectively at FOT (Fig. 4A–C). During the investigation period, 375.55 and 491.12 μg water accumulated per pair of ZS97A and ZS97B lodicules, respectively; however, the dry mass of ZS97A and ZS97B lodicules only increased by 34.12 and 43.07 μg pair^−1^, respectively. This result demonstrates that >91% of the increase in lodicule fresh weight was due to water. Moreover, there were positive correlations (*R*^2^>0.99) between lodicule fresh weight, lodicule volume, and water weight in the lodicule (Fig. 4D–F). Thus lodicule expansion mainly occurred due to water accumulation in the lodicule.

The accumulation of water in ZS97A lodicules was much less and slower than that in ZS97B lodicules (Fig. 4C). No clear difference in water weights was observed between ZS97A and ZS97B lodicules when the investigation began (0 h). However, water weight reached the maximum (515.16 μg pair^−1^) in ZS97B lodicules 98 h later, whereas water weight in ZS97A lodicules was only 99.47 μg pair^−1^ at the same time. The ZS97A lodicules continued to accumulate water for another 26 h. Moreover, the maximum water weight of the ZS97A lodicules was less than that of ZS97B at FOT (Fig. 4C). These results indicate that water accumulation in ZS97A lodicules was delayed and decreased significantly, which, in turn, delayed and inhibited lodicule expansion.

### Delayed water accumulation is related to JA deficiency in ZS97A lodicules

**Fig. 5.**
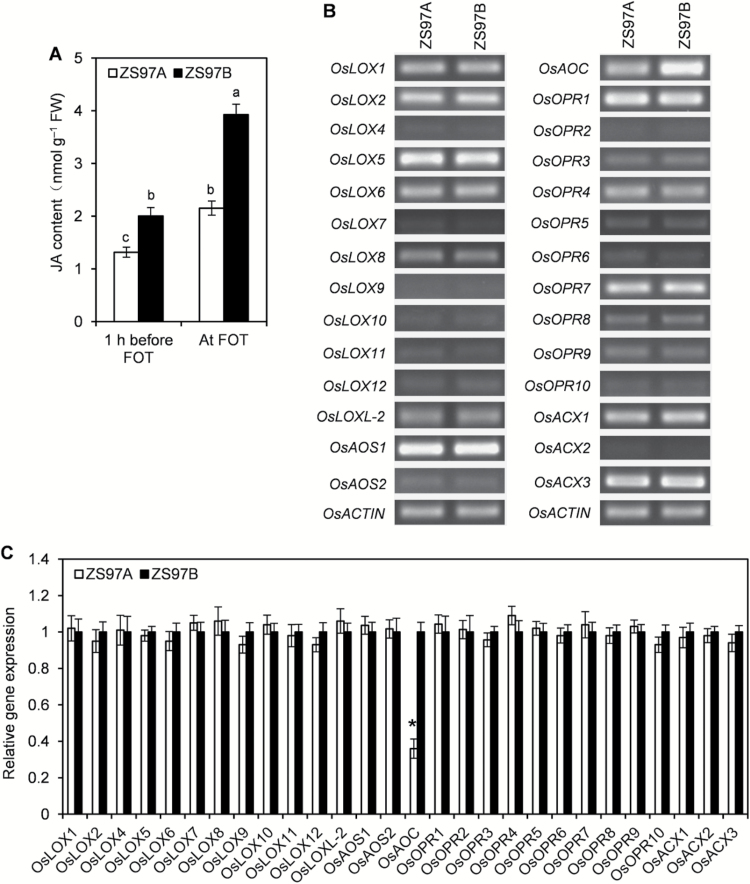
Comparison of jasmonic acid (JA) content and JA biosynthetic gene expression between ZS97A and ZS97B lodicules. At 10.00 h ZS97A and ZS97B florets that will open in 1 h and at 11.00 h (FOT) the opening florets of ZS97A and ZS97B were selected for lodicule collection. (A) Comparison of JA content between ZS97A and ZS97B lodicules 1 h before FOT and at FOT. Data are presented as means±SE. Standard errors of JA content were calculated from three biological replicates. Significant differences (*P*<0.05) are indicated by different letters. FOT, floret opening time; FW, fresh weight. (B, C) Comparison of JA biosynthetic gene expression between ZS97A and ZS97B lodicules at FOT by reverse transcription–polymerase chain reaction (RT-PCR) and quantitative RT-PCR analysis. The lodicules used for RT-PCR and qRT-PCR analysis were collected from opening florets at 11.00 h. Data are presented as means±SE (C). Three biological replicates with three technical replicates were each included for statistical analysis and error range analysis (C). Asterisk indicates significant difference (*P*<0.05) of gene expression in ZS97A lodicules compared with ZS97B lodicules (C).

Jasmonates play important roles regulating floret development, lodicule expansion, and lodicule wither (Cai *et al.*, 2014; Xiao *et al.*, 2014). JA contents were analysed in ZS97A and ZS97B lodicules to clarify whether retarded lodicule expansion was correlated with JA deficiency in ZS97A lodicules. JA content in ZS97A lodicules was significantly lower than that in ZS97B lodicules before FOT and at FOT (Fig. 5A), demonstrating that ZS97A lodicules were JA deficient. To uncover the cause of the JA deficiency, the expression levels of key genes involved in the JA biosynthetic pathway were compared between ZS97A and ZS97B lodicules. Although no obvious differences were detected in the expression levels of JA biosynthetic genes (including *OsLOX*, *OsAOS*, *OsOPR*, and *OsACX*) between ZS97A and ZS97B lodicules, expression of *OsAOC* was significantly down-regulated in ZS97A lodicules (Fig. 5B, C). This result suggests that the JA deficiency in ZS97A lodicules may have been caused by a decrease in JA biosynthesis via down-regulation of *OsAOC* expression.

**Fig. 6.**
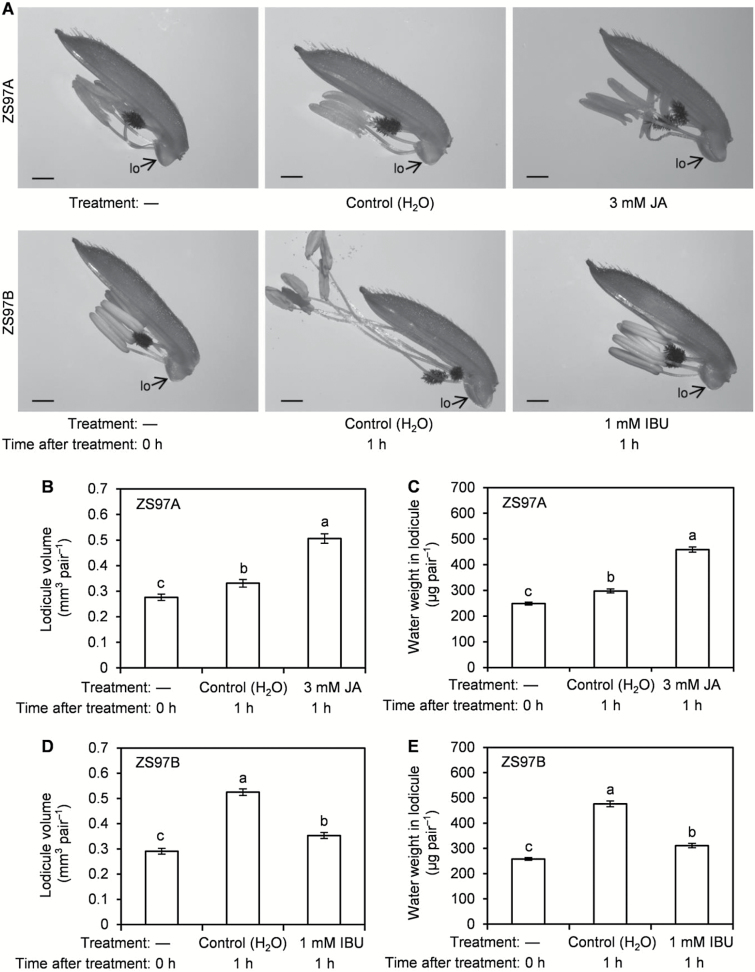
Effects of jasmonic acid (JA) and the JA biosynthetic inhibitor ibuprofen (IBU) on lodicule expansion and lodicule water accumulation. Panicles having florets nearing anthesis, in which several florets had flowered before the experimental date, were selected for tests. At 10.00 h (the beginning of treatment (0 h)), the selected panicles were treated with H_2_O (control), 3 mM JA or 1 mM IBU. At the beginning of the treatments (0 h) and 1 h after the treatments (11.00 h), the lodicules were collected and immediately photographed. Lodicule volume and water weight in lodicule of the samples were determined. All data were obtained from the upper florets of the treated panicles. (A) Effects of JA and IBU on lodicule expansion. (B, C) Effects of JA on ZS97A lodicule volume and water weight in ZS97A lodicule. (D, E) Effects of IBU on ZS97B lodicule volume and water weight in ZS97B lodicule. Scale bars (A): 1 mm. fi, filament; lo, lodicule. Data are presented as means±SE (B–E). Standard errors of lodicule volume and water weight in lodicule were calculated from ten and three biological replicates, respectively (B–E). Significant differences (*P*<0.05) are indicated by different letters (B–E). —, no treatment.

No differences in lodicule volume or water weight were observed between ZS97A and ZS97B at the beginning of the treatment (Fig. 6A–E). Applying JA strongly and quickly induced expansion of lodicules by promoting water accumulation in ZS97A lodicules, and lodicule volume and water weight in JA-treated lodicules were 1.53 and 1.54 times those of ZS97A control lodicules, respectively, after 1 h of treatment (Fig. 6A–C). By contrast, treatment with the JA biosynthetic inhibitor IBU inhibited water accumulation significantly in ZS97B lodicules, and lodicule volume and water weight in IBU-treated lodicules decreased 1.49- and 1.53-fold, respectively, compared with those of control ZS97B lodicules after 1 h of treatment (Fig. 6A, D, E). This result suggests that a particular JA concentration was required for water to accumulate rapidly in the lodicules, and that JA deficiency retarded water accumulation in ZS97A lodicules.

### JA deficiency retards the accumulation of osmotic regulation substances in ZS97A lodicules

**Fig. 7.**
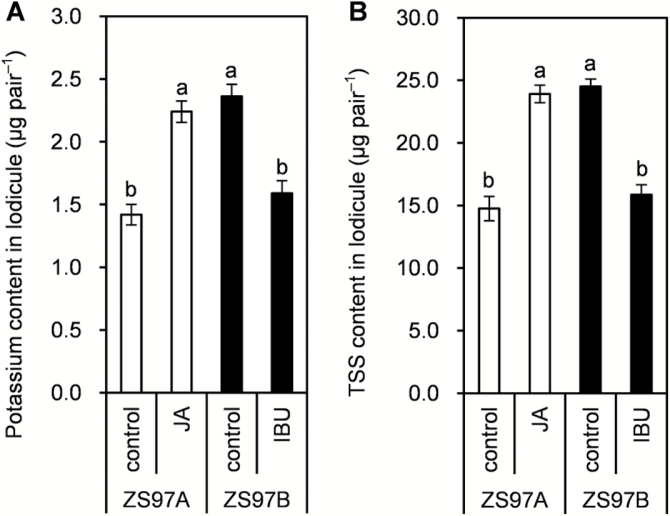
The effects of jasmonic acid (JA) on potassium and total soluble sugar (TSS) accumulation in lodicules. Panicles having florets nearing anthesis, in which several florets had flowered before the experimental date, were selected for tests. At 10.00 h (the beginning of treatment), the selected panicles were treated with control (H_2_O), 3 mM JA or 1 mM ibuprofen (IBU). At 11.00 h (1 h after treatment), lodicules from the upper florets of the treated panicles were collected for the determination of potassium and TSS contents. (A) The effects of JA and IBU on potassium content in lodicules at FOT. (B) The effects of JA and IBU on TSS content in lodicules at FOT. Data are presented as means±SE (A, B). Standard errors of potassium and TSS contents were calculated from three biological replicates, and significant differences (*P*<0.05) are indicated by different letters (A, B).

It has been reported that rapid water accumulation in lodicules is caused by an increase in osmotic pressure via the rapid accumulation of osmotic regulation substances (ORSs) in lodicule cells, and that potassium (K) and soluble sugars are the main ORSs in lodicule cells (Schuster, 1910; Wang *et al.*, 1991; Heslop-Harrison and Heslop-Harrison, 1996; Zeng *et al.*, 2004). K and TSS contents of control ZS97A lodicules were lower than those of ZS97B control lodicules (Fig. 7A, B). Applying JA strongly and quickly promoted the accumulation of K and TSS in ZS97A lodicules, and the contents were 1.58 and 1.62 times, respectively, those of ZS97A control lodicules after 1 h of JA treatment (Fig. 7A, B). On the other hand, IBU inhibited the accumulation of K and TSS in ZS97B lodicules (Fig. 7A, B). This result suggests that a particular level of JA was required for ORSs to rapidly accumulate in the lodicules; thus, JA deficiency retarded the accumulation of ORSs in lodicules, which led to the retardation of water accumulation in ZS97A lodicules.

### Scattered FOT in ZS97A is restored by JA

**Fig. 8.**
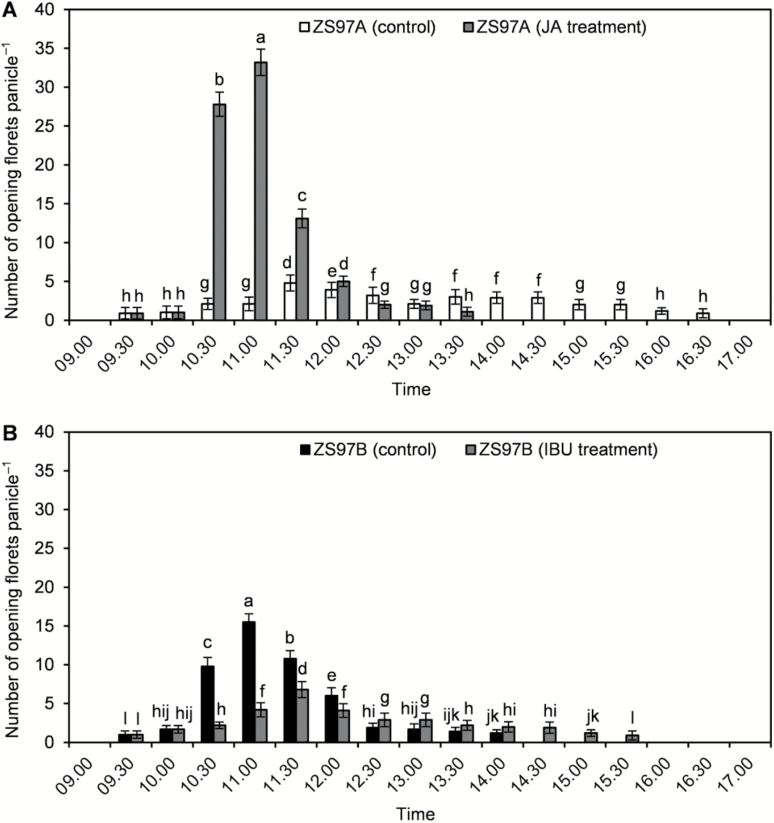
Effects of jasmonic acid (JA) and ibuprofen (IBU) on floret opening. Panicles having florets nearing anthesis, in which several florets had flowered before the experimental date, were selected for tests. At 10.00 h, the selected panicles were treated with control (H_2_O), 3 mM JA or 1 mM IBU. The number of florets opening on treated panicles was recorded at 09.00, 09.30, 10.00, 10.30, 11.00, 11.30, 12.00, 12.30, 13.00, 13.30, 14.00, 14.30, 15.00, 15.30, 16.00, 16.30, and 17.00 h. (A) Effect of JA on ZS97A floret opening. (B) Effect of IBU on ZS97B floret opening. Data are presented as means±SE (A, B). Standard errors of the number of opening florets per panicle were calculated from ten biological replicates, and significant differences (*P*<0.05) are indicated by different letters (A, B).

Applying JA strongly and quickly induced ZS97A florets to open, whereas applying IBU significantly inhibited opening of ZS97B florets (Fig. 8A, B). The FOT of ZS97A control florets was scattered, whereas JA-treated ZS97A florets revealed a clear flowering peak, as 86.15% of the JA-treated florets opened from 10.30 to 11.30 h, and 94.19% opened before 12.00 h. By contrast, the control florets of ZS97B had an obvious flowering peak, while the flowering peak of IBU-treated ZS97B florets was delayed and lower, as only 38.82% of the JA-treated florets opened from 10.30 to 11.30 h and 58.82% of the JA-treated florets opened before 12.00 h. These results demonstrate that JA restored scattered FOT in ZS97A by strongly and quickly inducing the florets to open, while IBU caused scattered FOT by inhibiting opening of ZS97B florets. These results indicate that JA plays a critical role regulating FOT, and that scattered FOT in ZS97A caused by JA deficiency can be restored by JA.

## Discussion

Scattered FOT in CMS rice leads to low rates of cross-pollination and low yields of hybrid rice seed production. However, until now, the mechanism of scattered FOT in CMS rice has remained elusive. Here, we provide evidence that scattered FOT in CMS rice ZS97A was caused by retarded expansion of lodicules via reduced water accumulation, which resulted from retarded ORS accumulation caused by JA deficiency in the lodicules.

### Scattered FOT is caused by retarded lodicule expansion in ZS97A florets

In male fertile rice ZS97B, the filaments extended and the lodicules swelled rapidly and simultaneously immediately before floret opening (Fig. 2A, D, E), suggesting that the extending filaments and/or swelling lodicules may provide the driving force to open florets by levering away the glumes. Filament extension and lodicule expansion were delayed and inhibited in ZS97A florets (Fig. 2A–E). Our result demonstrated that the expanding lodicules rather than the extending filaments were the driving force for floret opening (Fig. 3C–F). Therefore, retarded lodicule expansion, rather than retarded filament extension, led to a lack of driving force, which caused scattered FOT in ZS97A. This result is consistent with previous reports in cleistogamous florets of monocotyledonous plants including rice and wheat. The stamens and filaments of the *spw1-cls* rice mutant are normal, but the lodicules are transformed homeotically to lodicule–glume mosaic organs, thereby engendering cleistogamy (Yoshida *et al.*, 2007). The lodicules of cleistogamous wheat florets fail to expand while the filaments still attempt to extend in the normal manner although trapped between the lemma and palea (Heslop-Harrison and Heslop-Harrison, 1996).

### Retarded lodicule expansion is caused by the retardation of water accumulation in ZS97A lodicules

Lodicule expansion was mainly contributed by water accumulation in the lodicules, and over 91% of the increase in lodicule fresh weight was due to water accumulation (Fig. 4A–C). There were positive correlations between lodicule fresh weight, lodicule volume and water weight in the lodicules (Fig. 4D–F). Water accumulation in the lodicules was significantly delayed and decreased in CMS rice ZS97A (Fig. 4C). Thus, retarded lodicule expansion was caused by the retardation of water accumulation in ZS97A lodicules.

### The retardation of water accumulation is caused by retarded accumulation of ORSs in ZS97A lodicules

Water accumulation in lodicule cells results from a sudden increase in osmotic pressure of the cell contents (Schuster, 1910; Zeng *et al.*, 2004). K is required in large amounts by plants and is widely known for its rapid action as an ORS (Fischer, 1971; Heslop-Harrison and Heslop-Harrison, 1996). Movement of water into lodicule cells in sorghum and maize is an osmotic response mainly governed by K content in the lodicule cells (Heslop-Harrison and Heslop-Harrison, 1996). In addition, sugar is another likely ORS involved in increasing osmotic pressure in lodicule cells (Wang *et al.*, 1991). It has been reported that the contents of extracted, fully turgid lodicules tastes sweet (Heslop-Harrison and Heslop-Harrison, 1996), and that sap from lodicule cells in the expansion zone produces a characteristic sugar reaction with Fehling’s solution (Züderell 1909). These results indicate that K and sugar may be the main ORSs involved in raising the osmotic pressure, which promoted water accumulation in the lodicules. ORS contents in ZS97A lodicules were lower than those in ZS97B lodicules (Fig. 7A, B). The exogenous JA treatment strongly and quickly promoted accumulation of ORSs and water in ZS97A lodicules (Figs 6C and 7A, B), whereas treatment with the JA biosynthetic inhibitor IBU retarded ORS and water accumulation in ZS97B lodicules (Figs 6E and 7A, B). These results suggest that the retardation of water accumulation in ZS97A lodicules may be caused by retarded accumulation of ORSs.

### ORS and water accumulation is retarded by JA deficiency in ZS97A lodicules

Our results demonstrate that ZS97A lodicules suffered from a serious JA deficiency resulting from decreased biosynthesis (Fig. 5A–C), and that the accumulation of water and ORSs was retarded in ZS97A lodicules (Figs 4C and 7A, B). The exogenous JA treatment strongly and quickly promoted the expansion of lodicules by inducing water accumulation via rapid accumulation of ORSs in ZS97A lodicules (Figs 6A–C and 7A, B). Conversely, the IBU treatment retarded lodicule expansion by retarding the accumulation of water and ORSs in ZS97B lodicules (Figs 6A, D, E and 7A, B). These results indicate that a particular quantity of JA is required for lodicules to expand, and that the JA deficiency retarded lodicule expansion by retarding water accumulation via the retardation of ORS accumulation in ZS97A lodicules. These results are consistent with a previous study reporting that the *osjar1-2* and *osjar1-3* JA-resistant mutants also show retarded lodicule swelling and withering (Xiao *et al.*, 2014).

### Decreased *OsAOC* gene expression leads to JA deficiency, which in turn causes scattered FOT in ZS97A

JA is synthesized through the octadecanoid pathway, in which linolenic acid is converted to JA by a process that begins in chloroplasts and ends in peroxisomes (Park *et al.*, 2002). Linolenic acid is converted to 12-oxo-phytodienoic acid in chloroplasts through a multi-step enzymatic process involving lipoxygenase (LOX), allene oxide synthase (AOS) and allene oxide cyclase (AOC). Biosynthesis of JA proceeds with the action of cytoplasmic 12-oxo-phytodienoic acid reductase (OPR), and three rounds of β-oxidation, which can be catalysed by acyl-CoA oxidase (ACX) in rice (Kim *et al.*, 2007). Of all the genes involved in JA synthesis, only *OsAOC* expression level differed between ZS97A and ZS97B lodicules and it was significantly down-regulated in ZS97A lodicules (Fig. 5B, C). Rice has only one gene encoding OsAOC, and a normal expression level of *OsAOC* is required for maintaining a normal content of JA (Riemann *et al.*, 2013). JA contents in *OsAOC* knockout mutants (*cpm2* and *hebiba*) and *OsAOC* knockdown transgenic rice (AOC-RNAi) are severely reduced compared with that of control rice (Riemann *et al.*, 2003, 2013; Yara *et al.*, 2008). These results suggest that the JA deficiency in ZS97A lodicules was caused by decreased JA biosynthesis via down-regulation of *OsAOC* expression. JA plays a critical role in regulating FOT in rice; the JA-deficient mutant and JA-resistant mutant exhibit scattered FOT in rice (Biswas *et al.*, 2003; Haga, *et al.*, 2008; Xiao *et al.*, 2014). Evidence has revealed that *OsAOC* expression level and JA content increased as FOT approached (Huang *et al.*, 2015), whereas after FOT, *OsAOC* expression level and JA content decreased quickly (He *et al.*, 2012). Our results demonstrated that the expression level of *OsAOC* and the content of JA in ZS97A lodicules were lower than that of ZS97B, and the FOT of ZS97A was scattered and can be restored by application of exogenous JA (Figs 5B, C and 8A). These results indicated that as FOT approaches, the expression level of *OsAOC* is positively correlated with JA content, a particular content of JA in lodicules is necessary for floret opening in a certain period of the daytime, and down-regulation of *OsAOC* will be accompanied by decreased JA content and scattered FOT.

In conclusion, JA deficiency inhibited lodicule expansion by retarding the accumulation of ORSs and water, leading to scattered FOT in CMS rice ZS97A. We provide evidence that scattered FOT is associated with retarded lodicule expansion in ZS97A, which is correlated with the retardation of ORS and water accumulation in ZS97A lodicules, and retarded ORS and water accumulation is related to JA deficiency in ZS97A lodicules. These findings provide a scientific basis for increasing hybrid rice seed production by improving the flowering habit of CMS rice.

## Supplementary data

Supplementary data are available at *JXB* online.

Table S1. Primers used in the reverse transcription–polymerase chain reaction (RT-PCR) analysis.

Table S2. Primers used in the quantitative reverse transcription–polymerase chain reaction (qRT-PCR) analysis.

## Supplementary Material

Supplementary_Tables_S1_S2

## Abbreviations:

CMS: cytoplasmic male sterile
FOT: floret opening time
FW: fresh weight
IBU: ibuprofen
JA: jasmonic acid
ORS: osmotic regulation substance
TSS: total soluble sugar
ZS97A: Zhenshan 97A
ZS97B: Zhenshan 97B

## References

[CIT0001] Agrawal GK, Jwa N-S, Agrawal SK, Tamogami S, Iwahashi H, Rakwal R 2003 Cloning of novel rice allene oxide cyclase (*OsAOC*): mRNA expression and comparative analysis with allene oxide synthase (*OsAOS*) gene provides insight into the transcriptional regulation of octadecanoid pathway biosynthetic genes in rice. Plant Science 164, 979–992.

[CIT0002] Biswas KK, Neumann R, Haga K, Yatoh O, Iino M 2003 Photomorphogenesis of rice seedlings: a mutant impaired in phytochrome-mediated inhibition of coleoptile growth. Plant & Cell Physiology 44, 242–254. 12668770 10.1093/pcp/pcg040

[CIT0003] Cai Q, Yuan Z, Chen M, Yin C, Luo Z, Zhao X, Liang W, Hu J, Zhang D 2014 Jasmonic acid regulates spikelet development in rice. Nature Communications 5, 3476. 10.1038/ncomms4476 24647160

[CIT0004] Caldelari D, Wang G, Farmer EE, Dong X 2011 Arabidopsis *lox3 lox4* double mutants are male sterile and defective in global proliferative arrest. Plant Molecular Biology 75, 25–33. 21052784 10.1007/s11103-010-9701-9

[CIT0005] Cheng H, Song S, Xiao L, Soo HM, Cheng Z, Xie D, Peng J 2009 Gibberellin acts through jasmonate to control the expression of *MYB21*, *MYB24*, and *MYB57* to promote stamen filament growth in *Arabidopsis*. PLoS Genetics 5, e1000440. 19325888 10.1371/journal.pgen.1000440 PMC2654962

[CIT0006] Cheng SH, Zhuang JY, Fan YY, Du JH, Cao LY 2007 Progress in research and development on hybrid rice: a super-domesticate in China. Annals of Botany 100, 959–966. 17704538 10.1093/aob/mcm121 PMC2759200

[CIT0007] Delseny M, Salses J, Cooke R 2001 Rice genomics: Present and future. Plant Physiology and Biochemistry 39, 323–334.

[CIT0008] Dhakarey R, Peethambaran PK, Riemann M 2016 Functional analysis of jasmonates in rice through mutant approaches. Plants 5, 15. 10.3390/plants5010015 PMC4844424 27135235

[CIT0009] Feys B, Benedetti CE, Penfold CN, Turner JG 1994 Arabidopsis mutants selected for resistance to the phytotoxin coronatine are male sterile, insensitive to methyl jasmonate, and resistant to a bacterial pathogen. The Plant Cell 6, 751–759. 12244256 10.1105/tpc.6.5.751 PMC160473

[CIT0010] Fischer RA 1971 Role of potassium in stomatal opening in the leaf of *Vicia faba*. Plant Physiology 47, 555–558. 16657659 10.1104/pp.47.4.555 PMC396725

[CIT0011] Guo HM, Li HC, Zhou SR, Xue HW, Miao XX 2014 *Cis*-12-oxo-phytodienoic acid stimulates rice defense response to a piercing-sucking insect. Molecular Plant 7, 1683–1692. 25239066 10.1093/mp/ssu098

[CIT0012] Haga K, Iino M 2004 Phytochrome-mediated transcriptional up-regulation of ALLENE OXIDE SYNTHASE in rice seedlings. Plant & Cell Physiology 45, 119–128. 14988482 10.1093/pcp/pch025

[CIT0013] Haga K, Kiyota S, Jikumaru Y, Kamiya Y, Takano M, Iino M 2008 Functional analysis of a rice allene oxide synthase gene (*OsAOS1*) that functions for jasmonate biosynthesis. Plant and Cell Physiology 49 (Suppl), P0364 (doi: 10.14841/jspp.2008.0.0364.0).

[CIT0014] Hazman M, Hause B, Eiche E, Nick P, Riemann M 2015 Increased tolerance to salt stress in OPDA-deficient rice ALLENE OXIDE CYCLASE mutants is linked to an increased ROS-scavenging activity. Journal of Experimental Botany 66, 3339–3352. 25873666 10.1093/jxb/erv142 PMC4449546

[CIT0015] He Y, Fukushige H, Hildebrand DF, Gan S 2002 Evidence supporting a role of jasmonic acid in Arabidopsis leaf senescence. Plant Physiology 128, 876–884. 11891244 10.1104/pp.010843 PMC152201

[CIT0016] He Y-M, Lin Y-J, Zeng X-C 2012 Dynamic changes of jasmonic acid biosynthesis in rice florets during natural anthesis. Acta Agronomica Sinica 38, 1891–1899.

[CIT0017] Heslop-Harrison Y, Heslop-Harrison JS 1996 Lodicule function and filament extension in the grasses: potassium ion movement and tissue specialization. Annals of Botany 77, 573–582.

[CIT0018] Hoshikawa K 1989 The growing rice plant. Tokyo: Nosan Gyoson Bunka Kyokai (Nobunkyo), 241–243.

[CIT0019] Huang J, Cai M, Long Q, Liu L, Lin Q, Jiang L, Chen S, Wan J 2014 OsLOX2, a rice type I lipoxygenase, confers opposite effects on seed germination and longevity. Transgenic Research 23, 643–655. 24792034 10.1007/s11248-014-9803-2

[CIT0020] Huang J-B, He Y-M, Zeng X-C, Xiang M-L, Fu Y-Q 2015 Changes of JA levels in floral organs and expression analysis of JA signaling genes in lodicules before floret opening in rice. Scientia Agricultura Sinica 48, 1219–1227.

[CIT0021] Ieggli CV, Bohrer D, do Nascimento PC, de Carvalho LM, Garcia SC 2010 Determination of sodium, potassium, calcium, magnesium, zinc, and iron in emulsified egg samples by flame atomic absorption spectrometry. Talanta 80, 1282–1286. 20006088 10.1016/j.talanta.2009.09.024

[CIT0022] Jia H, Zhang C, Pervaiz T 2016 Jasmonic acid involves in grape fruit ripening and resistant against *Botrytis cinerea*. Functional & Integrative Genomics 16, 79–94. 26498957 10.1007/s10142-015-0468-6

[CIT0023] Kim MC, Kim TH, Park JH, Moon BY, Lee CH, Cho SH 2007 Expression of rice acyl-CoA oxidase isoenzymes in response to wounding. Journal of Plant Physiology 164, 665–668. 17000027 10.1016/j.jplph.2006.08.003

[CIT0024] Kuroda H, Oshima T, Kaneda H, Takashio M 2005 Identification and functional analyses of two cDNAs that encode fatty acid 9-/13-hydroperoxide lyase (CYP74C) in rice. Bioscience, Biotechnology, and Biochemistry 69, 1545–1554. 10.1271/bbb.69.1545 16116284

[CIT0025] Lee SH, Sakuraba Y, Lee T, Kim KW, An G, Lee HY, Paek NC 2015 Mutation of *Oryza sativa CORONATINE INSENSITIVE 1b* (*OsCOI1b*) delays leaf senescence. Journal of Integrative Plant Biology 57, 562–576. 25146897 10.1111/jipb.12276

[CIT0026] Liu H, Li X, Xiao J, Wang S 2012 A convenient method for simultaneous quantification of multiple phytohormones and metabolites: application in study of rice-bacterium interaction. Plant Methods 8, 2. 22243810 10.1186/1746-4811-8-2 PMC3274484

[CIT0027] Liu L, Li H, Zeng H, Cai Q, Zhou X, Yin C 2016 Exogenous jasmonic acid and cytokinin antagonistically regulate rice flag leaf senescence by mediating chlorophyll degradation, membrane deterioration, and senescence-associated genes expression. Journal of Plant Growth Regulation 35, 366–376.

[CIT0028] Marla SS, Singh VK 2012 *LOX* genes in blast fungus (*Magnaporthe grisea*) resistance in rice. Functional & Integrative Genomics 12, 265–275. 22370743 10.1007/s10142-012-0268-1

[CIT0029] Mizuno K, Iida T, Takano A, Yokoyama M, Fujimura T 2003 A new 9-lipoxygenase cDNA from developing rice seeds. Plant & Cell Physiology 44, 1168–1175. 14634153 10.1093/pcp/pcg142

[CIT0030] Ohta H, Shirano Y, Tanaka K, Morita Y, Shibata D 1992 cDNA cloning of rice lipoxygenase L-2 and characterization using an active enzyme expressed from the cDNA in *Escherichia coli*. European Journal of Biochemistry 206, 331–336. 1597177 10.1111/j.1432-1033.1992.tb16931.x

[CIT0031] Pan X, Welti R, Wang X 2008 Simultaneous quantification of major phytohormones and related compounds in crude plant extracts by liquid chromatography-electrospray tandem mass spectrometry. Phytochemistry 69, 1773–1781. 18367217 10.1016/j.phytochem.2008.02.008

[CIT0032] Park J-H, Halitschke R, Kim HB, Baldwin IT, Feldmann KA, Feyereisen R 2002 A knock-out mutation in allene oxide synthase results in male sterility and defective wound signal transduction in *Arabidopsis* due to a block in jasmonic acid biosynthesis. The Plant Journal 31, 1–12. 12100478 10.1046/j.1365-313x.2002.01328.x

[CIT0033] Peng Y-L, Shirano Y, Ohta H, Hibino T, Tanaka K, Shibata D 1994 A novel lipoxygenase from rice. The Journal of Biological Chemistry 269, 3755–3761. 7508918

[CIT0034] Riemann M, Dhakarey R, Hazman M, Miro B, Kohli A, Nick P 2015 Exploring jasmonates in the hormonal network of drought and salinity responses. Frontiers in Plant Science 6, 1077. 26648959 10.3389/fpls.2015.01077 PMC4665137

[CIT0035] Riemann M, Haga K, Shimizu T 2013 Identification of rice *Allene Oxide Cyclase* mutants and the function of jasmonate for defence against *Magnaporthe oryzae*. The Plant Journal 74, 226–238. 23347338 10.1111/tpj.12115

[CIT0036] Riemann M, Muller A, Korte A, Furuya M, Weiler EW, Nick P 2003 Impaired induction of the jasmonate pathway in the rice mutant *hebiba*. Plant Physiology 133, 1820–1830. 14605232 10.1104/pp.103.027490 PMC300735

[CIT0037] Sanders PM, Lee PY, Biesgen C, Boone JD, Beals TP, Weiler EW, Goldberg RB 2000 The arabidopsis *DELAYED DEHISCENCE1* gene encodes an enzyme in the jasmonic acid synthesis pathway. The Plant Cell 12, 1041–1061. 10899973 10.1105/tpc.12.7.1041 PMC149048

[CIT0038] Schuster J 1910 Uber die Morphologie der Grasblüte. Flora 100, 213–266.

[CIT0039] Sobajima H, Takeda M, Sugimori M 2003 Cloning and characterization of a jasmonic acid-responsive gene encoding 12-oxophytodienoic acid reductase in suspension-cultured rice cells. Planta 216, 692–698. 12569412 10.1007/s00425-002-0909-z

[CIT0040] Song P, Xia K, Wu C-W, Bao D-P, Chen L-L, Zhou X, Cao X-Z 2001 Differential response of floret opening in male-sterile and male-fertile rices to methyl jasmonate. Acta Botanica Sinica 43, 480–485.

[CIT0041] Song S, Qi T, Huang H, Xie D 2013 Regulation of stamen development by coordinated actions of jasmonate, auxin, and gibberellin in *Arabidopsis*. Molecular Plant 6, 1065–1073. 23543439 10.1093/mp/sst054

[CIT0042] Song S, Qi T, Wasternack C, Xie D 2014 Jasmonate signaling and crosstalk with gibberellin and ethylene. Current Opinion in Plant Biology 21, 112–119. 25064075 10.1016/j.pbi.2014.07.005

[CIT0043] Stintzi A, Browse J 2000 The *Arabidopsis* male-sterile mutant, *opr3*, lacks the 12-oxophytodienoic acid reductase required for jasmonate synthesis. Proceedings of the National Academy of Sciences, USA 97, 10625–10630. 10.1073/pnas.190264497 PMC27075 10973494

[CIT0044] Tani T, Sobajima H, Okada K 2008 Identification of the *OsOPR7* gene encoding 12-oxophytodienoate reductase involved in the biosynthesis of jasmonic acid in rice. Planta 227, 517–526. 17938955 10.1007/s00425-007-0635-7

[CIT0045] von Malek B, van der Graaff E, Schneitz K, Keller B 2002 The *Arabidopsis* male-sterile mutant *dde2-2* is defective in the *ALLENE OXIDE SYNTHASE* gene encoding one of the key enzymes of the jasmonic acid biosynthesis pathway. Planta 216, 187–192. 12430030 10.1007/s00425-002-0906-2

[CIT0046] Wang R, Shen W-B, Jiang L, Liu L-H, Zhai H-Q, Wan J-M 2008 Prokaryotic expression, purification and characterization of a novel rice seed lipoxygenase gene *OsLOX1*. Chinese Journal of Rice Science 22, 118–124.

[CIT0047] Wang S, Ichii M, Taketa S, Xu L, Xia K, Zhou X 2002 Lateral root formation in rice (*Oryza sativa*): promotion effect of jasmonic acid. Journal of Plant Physiology 159, 827–832.

[CIT0048] Wang Z, Gu Y, Gao Y 1989 Studies on the mechanism of rice glume opening: II. effect of CO_2_ on glume-opening. Acta Agronomica Sinica 15, 59–66.

[CIT0049] Wang Z, Gu Y, Gao Y 1991 Studies on the mechanism of the anthesis of rice: III. Structure of the lodicule and changes of its contents during flowering. Acta Agronomica Sinica 17, 96–101.

[CIT0050] Xiao Y, Chen Y, Charnikhova T 2014 OsJAR1 is required for JA-regulated floret opening and anther dehiscence in rice. Plant Molecular Biology 86, 19–33. 24947835 10.1007/s11103-014-0212-y

[CIT0051] Xu H, Wei Y, Zhu Y 2015 Antisense suppression of *LOX3* gene expression in rice endosperm enhances seed longevity. Plant Biotechnology Journal 13, 526–539. 25545811 10.1111/pbi.12277

[CIT0052] Yara A, Yaeno T, Hasegawa M, Seto H, Seo S, Kusumi K, Iba K 2008 Resistance to *Magnaporthe grisea* in transgenic rice with suppressed expression of genes encoding allene oxide cyclase and phytodienoic acid reductase. Biochemical and Biophysical Research Communications 376, 460–465. 18786507 10.1016/j.bbrc.2008.08.157

[CIT0053] Ye M, Song Y, Long J 2013 Priming of jasmonate-mediated antiherbivore defense responses in rice by silicon. Proceedings of the National Academy of Sciences, USA 110, E3631–E3639. 10.1073/pnas.1305848110 PMC3780902 24003150

[CIT0054] Yin C, Gan L, Ng D, Zhou X, Xia K 2007 Decreased panicle-derived indole-3-acetic acid reduces gibberellin A1 level in the uppermost internode, causing panicle enclosure in male sterile rice Zhenshan 97A. Journal of Experimental Botany 58, 2441–2449. 17556768 10.1093/jxb/erm077

[CIT0055] Yoshida H, Itoh J, Ohmori S 2007 *superwoman1-cleistogamy*, a hopeful allele for gene containment in GM rice. Plant Biotechnology Journal 5, 835–846. 17764519 10.1111/j.1467-7652.2007.00291.x

[CIT0056] Yuan Z, Zhang D 2015 Roles of jasmonate signalling in plant inflorescence and flower development. Current Opinion in Plant Biology 27, 44–51. 26125498 10.1016/j.pbi.2015.05.024

[CIT0057] Zeng X, Zhou X, Wu X 2004 Advances in study of opening mechanism in rice florets. Scientia Agricultura Sinica 37, 188–195.

[CIT0058] Zeng X, Zhou X, Zhang W, Murofushi N, Kitahara T, Kamuro Y 1999 Opening of rice floret in rapid response to methyl jasmonate. Journal of Plant Growth Regulation 18, 153–158. 10688703 10.1007/pl00007063

[CIT0059] Zhang H, Xu C, He Y 2013 Mutation in *CSA* creates a new photoperiod-sensitive genic male sterile line applicable for hybrid rice seed production. Proceedings of the National Academy of Sciences, USA 110, 76–81. 10.1073/pnas.1213041110 PMC3538234 23256151

[CIT0060] Zhang X, Jiang D, Zheng C, Dai T, Cao W 2011 Post-anthesis salt and combination of salt and waterlogging affect distributions of sugars, amino acids, Na^+^ and K^+^ in wheat. Journal of Agronomy and Crop Science 197, 31–39.

[CIT0061] Zhou G, Qi J, Ren N, Cheng J, Erb M, Mao B, Lou Y 2009 Silencing *OsHI-LOX* makes rice more susceptible to chewing herbivores, but enhances resistance to a phloem feeder. The Plant Journal 60, 638–648. 19656341 10.1111/j.1365-313X.2009.03988.x

[CIT0062] Zhou G, Ren N, Qi J, Lu J, Xiang C, Ju H, Cheng J, Lou Y 2014 The 9-lipoxygenase Osr9-LOX1 interacts with the 13-lipoxygenase-mediated pathway to regulate resistance to chewing and piercing-sucking herbivores in rice. Physiologia Plantarum 152, 59–69. 24410960 10.1111/ppl.12148

[CIT0063] Züderell H 1909 Uber das Auflblühen der Gräser. Sitzungsberichte Osterreichische Akademie der Wissenschaften, Mathematisch. Wissenschaftliche Klasse Abteilung 1 118, 1402–1426.

