# Supplementary material for: Jasmonic acid deficiency leads to scattered floret opening time in cytoplasmic male sterile rice Zhenshan 97A
Source: J Exp Bot. 2017 Jul 26;68(16):4613–25. doi: 10.1093/jxb/erx251 (PMC5853226; doi:10.1093/jxb/erx251)
Supplement: Supplementary_Tables_S1_S2 [file erx251_suppl_supplementary_tables_s1_s2.pdf]

JA deficiency leads to scattered floret opening time in cytoplasmic male sterile rice Zhenshan 97A. Li Liu, Zhengshan Zou, Ke Qian, Chan Xia, Ying He, Hanlai Zeng, Xie Zhou, Michael Riemann, and Changxi Yin

SUPPLEMENTARY DATA

Table S1. Primers used in the reverse transcription-polymerase chain reaction (RT-PCR) analysis

Gene	Forward primer 5' → 3'	Reverse primer 5' → 3'
OsLOX1	CCTCCACCTCCACCAACATC	GGAGGAACACGTCGTGGTAG
OsLOX2	CATTGATCCCATCGCTGATT	GAGCCATGCAAACTTGTCCT
OsLOX4	TGTGATGGAGCCGTTCGTGA	CGCCGTCGTCGGTGTAGTAG
OsLOX5	CCAACCAGACAAAGGCAGTA	GGGAGAACACCCTCAACAATA
OsLOX6	TCCGCTACGACACCCCCGA	TACAGCCTGCTCCCCTCCA
OsLOX7	CATTCAACAGGCGATGGAGCA	GACATGAGTCTTGGCGAGGTG
OsLOX8	CTTCGCCAAGGCTTATCTGC	TAAGGGAACTGCTTGCTGCC
OsLOX9	GAGGAAGGGCAACGTGTAC	GTCTCTGAGCCATGCGAAC
OsLOX10	TGCTCTTCCACCGCAACGAT	TGGCGAACGGCTCCATCACC
OsLOX11	CCATCAAGGAGTGGGTGTCA	GGGGCGATTGGGGAAGTAGC
OsLOX12	CCGTCAGCGTCGGACATTTA	GGAACGTGTCCAGCAAAACC
OsLOXL-2	AAGTATCGCTACAACCGCGT	GTGTGAGGTCGACGTAGGTG
OsAOS1	CTACGGGTACGAGGTGAGGGA	GGTGACGGTGACAGATGAGCC
OsAOS2	GCCGCTCTGGAGAAGATGGA	ACGATTGACGGCGGAGGTT
OsAOC	ACAGCGGAAGCCTGGAC	GGGATGCCCTTGAGGTAGA
OsOPR1	GGGAGGAAGGGAACAAGGTG	GGGTAATCTGTATAGCCAACAACG
OsOPR2	CCACTGTCATCTCCCCCAC	CACCATGCCTGAATCGTCC
OsOPR3	GCAACTCAACAAGCACCCAGA	GCCAAGAAGAGCCTCCCATAG
OsOPR4	GTGGCTCTCGGCTCGTACAT	CGCCCTCGTCTTTCTCATCA
OsOPR5	GAGCAGCTCAACAAGCACGAG	GCCAAGAAAAGCCTTCCAAAG
OsOPR6	CTGACACCGTACAAGCAGG	TTGAGATGGGAGCCAATCC
OsOPR7	CCAAGGGAGGCATCTTTTTC	CATACTCGTCAGTGCGGTCA
OsOPR8	CCCCAAGGAGGCTAAGAGAAG	TGAGTATGGCGAGAGACGGAC
OsOPR9	TCATTGTGAACGGAGGGTACG	TGCTCAAGAAACGGGTAGTCC
OsOPR10	CGCTGGTGATTTTAAGGATG	GTAGAGGACGCCGAGGTTGT
OsACX1	TCACAAGTTACGAAAGAGGGAA	CTCACCCACAAACCTAAATGC
OsACX2	CAGCCTCATGGCGAACGA	TTGTAGGGCCTGGACATTAGATC
OsACX3	GACCACTCCCTCGCCATCA	CATCCGGCAAAACATCAGC
OsACTIN	GCGATAATGGAACTGGTATGG	GTTGAGAGGAGCCTCGGTGAG

Table S2. Primers used in the quantitative reverse transcription-polymerase chain reaction (qRT-PCR) analysis

Gene	Forward primer 5' → 3'	Reverse primer 5' → 3'
OsLOX1	CTCGTGGTTCAGAGACGAGG	TGCTCACGATGGGAAACTCC
OsLOX2	CGATGAGGCATTTGAGGAGC	TGTGGTGGAAGCCTTGGAAA
OsLOX4	TCAAACAGGACAAGGAGGCG	AATTCCGTGAGACGCGTGAT
OsLOX5	ACGAGAAATGCTCGCAGGAG	GGCCCTCCATGTTGTGCTTA
OsLOX6	ATCAAGAAGGACAAGTTCGCGT	TGGAGACGCTCGATGTTGAC
OsLOX7	TCTGACCATTCAACAGGCGAT	CACCCCCTCCGTGTTTATCC
OsLOX8	CAACATCAGGGATCTCATCGG	ATGCACCGTTGCCTTCTTCTT
OsLOX9	GACCCTAAGTCGGAGACGAG	GTCTTGCGGAGGAAGTAGTC
OsLOX10	GACCACCACGACCACTTCAT	TAGGCTGAGCTCGATTGCTG
OsLOX11	GGTCATGACAGCAGAGGAGG	CCTCCGTCAGGAAGAAGAGC
OsLOX12	AATGGCATTCCATGGGGAGG	TCACATGGTCTGACACCCAC
OsLOXL-2	ACCCCAACAATGGGAACAGG	CGGGTATGCCCATCTTCTCC
OsAOS1	ATCACCAAGTGGGTGCTGTT	GGCGGTCGTAGTCCTTCTTC
OsAOS2	CGTTGACAACAAGCAGTGCC	CGGAGGTTGAAGCTTTGGTG
OsAOC	GCTCAACCAGATCGTCTTCCC	TTGTTGAGGCAGGCGTGG
OsOPR1	TCAATGGCACTTTCATCGCTG	CCTCGGCAAGTCAGGGTTAGC
OsOPR2	CAGGCTTTCTCTACTGCCACAT	GCCACCACCTTGTTCCCTT
OsOPR3	AGGTCAAGGCGTGGAAACC	GTAGTTACCGTTGGTGGAGGC
OsOPR4	GCTGTCACCGTTCATCGACT	GCCATTCTTGGCTCCACCAT
OsOPR5	CTTTTCTTGGCGAACCCTGAC	AGCAACCGAGTTGTTCTGATCC
OsOPR6	CGATCATCAGGCTATCCCGC	CACGTTGCCGTAGGATCGG
OsOPR7	CTGCCGGTTCCTACTTGAGG	GCGTCAAGGTGATCAATGGC
OsOPR8	AAGTACGGAGGAAGCATCGC	TGAGTATGGCGAGAGACGGA
OsOPR9	CCTGGCTTTCTTTTCGGCAC	TAGGCGTGACATGAACCACC
OsOPR10	ATGGATGCAATCAGTCCGCT	TGCTTGTCCGTGCTTGAGAT
OsACX1	GAGCAGCTGGGAAAGCTGTA	TACAGCGCCGGGTAAACATT
OsACX2	GGCGTCCAGTACAGCCTTT	ATCCATGGTGCAGCTCTGTC
OsACX3	AAGGTTTGAAGACAGAGAATCGCAT	GCATTAGAACATTATTGTCGCCCT
OsACTIN	CTGACGGAGCGTGGTTACTCAT	TCATAGTCCAGGGCGATGTAGG
